# Case report of clostridium difficile infection after rectal resection with ileostomy

**DOI:** 10.1186/s12957-025-03713-5

**Published:** 2025-03-01

**Authors:** Hongwei Guo, Huiyuan Jiang, Haiyi Liu

**Affiliations:** Department of Colorectal Surgery, Shanxi Cancer Hospital, ZhiGongXinCun Street No.3, XinHuaLing District, Taiyuan, Shanxi 030013 China

**Keywords:** Colorectal cancer, Ileostomy, Clostridium difficile infection, Fecal microbiota transplantation

## Abstract

Colorectal cancer is the third most common cancer worldwide, with high incidence and mortality rates. Surgical resection is the primary treatment for rectal cancer. To reduce the occurrence and severity of postoperative complications such as anastomotic leakage, prophylactic ileostomy is often performed concurrently. However, following ileostomy creation, there is a disruption in intestinal ecology, making patients susceptible to clostridium difficile infection. clostridium difficile is a Gram-positive anaerobic spore-forming bacterium that is resistant to most antibiotics due to spore formation, leading to high recurrence rates and treatment failure. Additionally, in the early stages of clostridium difficile infection, increased ileostomy output can be challenging to differentiate from normal postoperative conditions, potentially resulting in missed diagnosis, delayed treatment, and increased healthcare burden.This case report describes a case of high out-put ileostomy caused by clostridium difficile infection following rectal resection with ileostomy, which was successfully treated by fecal microbiota transplantation, providing evidence-based medicine for clinical practice.

## Introduction

Colorectal cancer (CRC) is the third most common cancer worldwide, with high mortality rates and an increasing trend. According to the 2020 China Cancer Report, the incidence and mortality rates of colorectal cancer in China are the second and fifth highest among all malignant tumors, respectively [1]. Surgical resection is the preferred treatment for colorectal cancer, and prophylactic ileostomy is increasingly becoming an essential component of curative resection for low rectal cancer due to its ability to reduce the incidence and severity of anastomotic leakage and other postoperative complications.However, the creation of a prophylactic ileostomy results in the long-term opening of the distal bowel segment, rendering it nonfunctional, leading to significant changes in the intestinal mucosa, smooth muscle, and microbial flora, resulting in gut microbial dysbiosis, and increasing the risk of gut infections [2]. A clinical study reported that the incidence rate of clostridium difficile infection (CDI) in patients undergoing end-ileostomy after total colectomy is as high as 16% [3].

Clostridium difficile (CD) is a Gram-positive anaerobic spore-forming bacterium, widely present in the environment, transmitted through the fecal-oral route, and is the most common pathogen causing hospital-acquired diarrhea and antibiotic-associated diarrhea [4, 5]. Clinically, approximately 25-33% of antibiotic-associated diarrhea and 90% of pseudomembranous colitis are caused by CDI [6]. Furthermore, due to the ability of Clostridium difficile to produce spores, and spores being resistant to most antibiotics, CDI is prone to recurrence, with a high treatment failure rate, posing a persistent problem in healthcare settings [7]. This case report describes a successful case of CDI treated with fecal microbiota transplantation following rectal cancer surgery with protective ileostomy, providing evidence-based medicine for the treatment of CDI after rectal resection with protective ileostomy.

## Case presentation

**Fig. 1:**
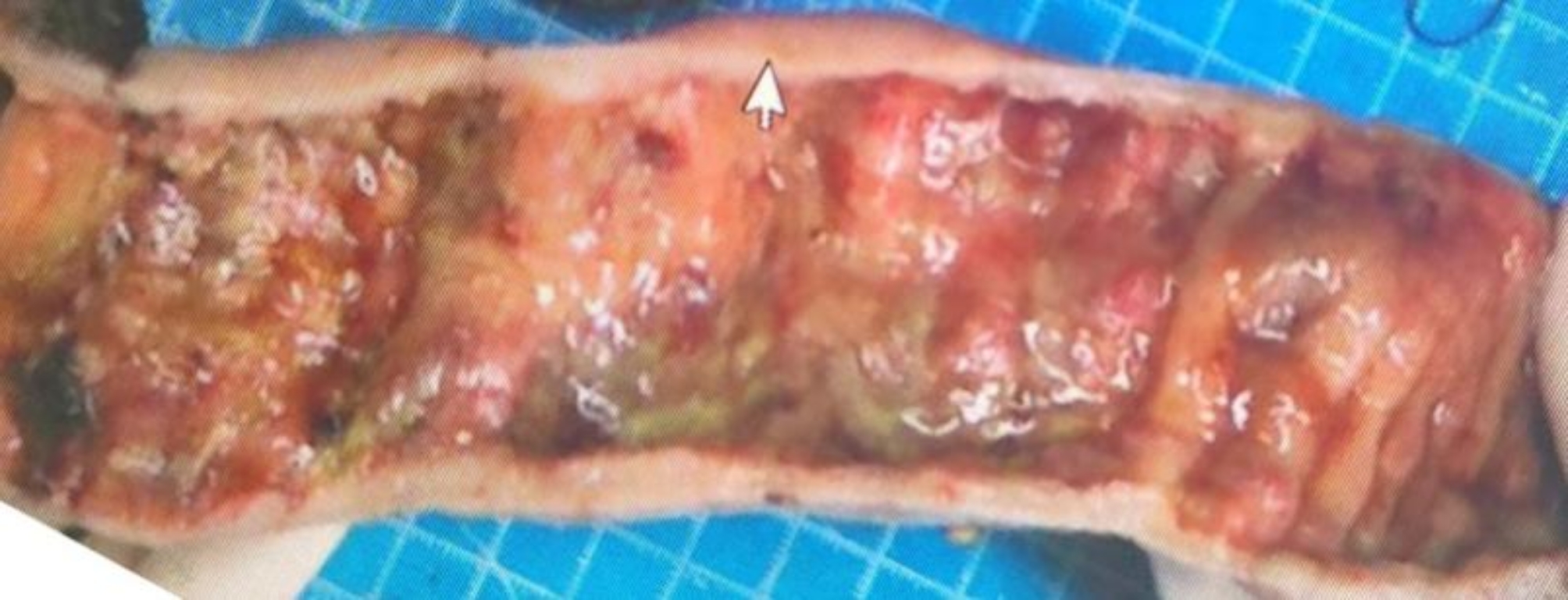
Resected small intestine (thickened intestinal wall, loss of folds)

**Fig. 2:**
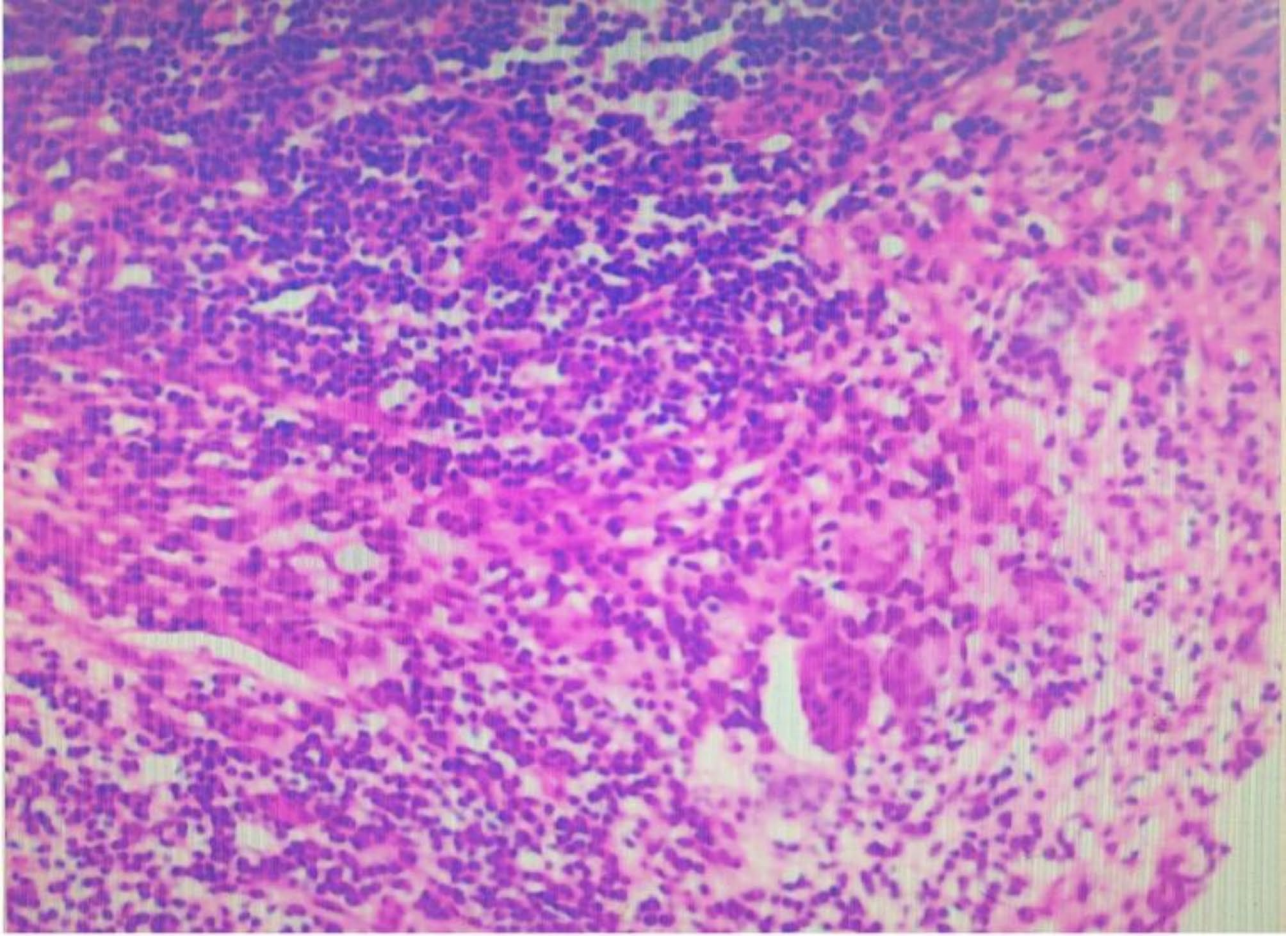
Small intestine H&E (Hematoxylin and Eosin) histological section

**Fig. 3:**
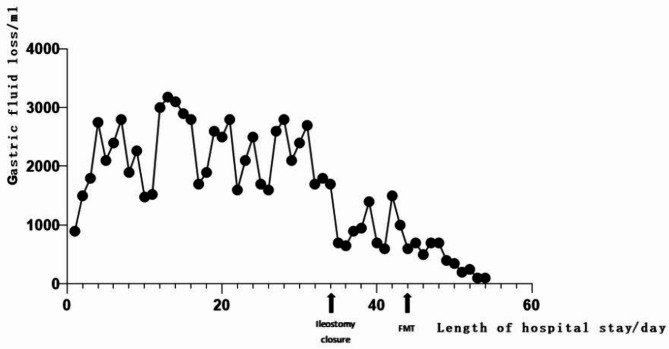
Changes in Digestive Fluid Loss During Hospitalization

The patient is a 65-year-old male with rectal adenocarcinoma and left renal clear cell carcinoma. He underwent rectal resection, partial left nephrectomy, and protective ileostomy, and was discharged smoothly 8 days postoperatively. Nine days after discharge, the patient was readmitted due to fever, nausea, vomiting, abdominal pain, distension, cessation of gas and stool passage after eating apples. On admission: temperature 38℃, heart rate 114/min, respiratory rate 34/min, blood pressure 60/40 mmHg; laboratory findings: white blood cell 2.93*10^9/L, neutrophil percentage 76.2%, red blood cell 3.83*10^12/L, hemoglobin 100 g/L, PCT 183.01 ng/mL, urea 21.96 mmol/L, creatinine 295 µmol/L, potassium 4.53 mmol/L, sodium 122 mmol/L, chloride 93 mmol/L, NT-ProBNP 3789 pg/mL. Abdominal CT scan: small bowel dilation with fluid and gas accumulation. Admission diagnosis: (1) Acute intestinal obstruction (2) Severe intestinal infection (3) Septic shock (4) Acute renal failure. The patient was transferred to the intensive care unit for shock and acute renal failure management, receiving aggressive fluid resuscitation to correct shock and antimicrobial therapy with imipenem and cilastatin sodium. Five days later, the symptoms of obstruction improved and the body temperature returned to normal, but then a high-volume ileostomy diarrhea occurred, and the diarrhea volume was more than 2000 ml/d, which did not significantly improve with treatments including somatostatin, omeprazole, montmorillonite, and loperamide. Based on our past clinical experience, patients with the high output of the ileostomy usually improve after ileostomy reversal surgery. Therefore, after 30 days of maintenance treatment, colonoscopy and lower gastrointestinal radiography were performed, which indicated that the rectal anastomotic stoma had healed well. Subsequently, ileostomy reversal surgery was carried out. Intraoperatively, intestinal edema, dilatation, loss of folds, and thickening of the intestinal wall were observed (Fig. 1). Pathological examination of the resected small intestine revealed chronic inflammation with erosion and intermittent shallow ulcers(Fig. 2). Despite the surgery, gastrointestinal function remained unrecovered, with a gastrointestinal decompression volume of approximately 1000 ml daily, accompanied by diarrhea occurring over 10 times per day. The stool was yellow-green, watery, and contained black residue. Stool culture for pathogenic bacteria suggested infection with clostridium difficile. Oral vancomycin was initiated followed by fecal microbiota transplantation after six doses of vancomycin, along with digestive juice reinfusion, oral Saccharomyces boulardii, glutamine granules, pectin, and probiotics to improve intestinal microbiota. The patient’s condition gradually improved after fecal microbiota transplantation therapy and was discharged 18 days later. The amount of gastrointestinal fluid loss during the hospitalization is shown in Fig. 3.The final diagnosis was Clostridium difficile-associated enterocolitis. At the 3-month follow-up post-discharge, the patient’s diet and bowel habits were satisfactory.

## Discussion

Clostridium difficile is a Gram-positive, anaerobic spore-forming bacillus that resides in the normal human gut flora. It is a common causative agent of healthcare-associated intestinal infections, leading to Clostridium difficile-associated diarrhea, Clostridium difficile-associated colitis, Clostridium difficile-associated enteritis, and pseudomembranous colitis. Under conditions of prolonged or inappropriate antibiotic use, compromised immunity, gastrointestinal surgery, and other risk factors, the gut microbiota becomes imbalanced, with a reduction in dominant gut flora such as Lactobacillus and Bifidobacterium, leading to decreased antagonistic effects against C. difficile. This allows for the proliferation of C. difficile and the production of toxins. These toxins cause enterocyte death, intestinal wall necrosis, fluid accumulation, and watery diarrhea. Necrotic enterocytes and inflammatory cells form a pseudomembrane on the intestinal mucosa, known as pseudomembranous colitis [8, 9].In severe cases, complications such as toxic megacolon, intestinal perforation, and septic shock can occur, endangering the patient’s life [10]. Additional risk factors for CDI include advanced age, male gender, proton pump inhibitor use, and frequent enemas [11]. The most common clinical manifestation of CDI is diarrhea, which may be accompanied by abdominal pain, fever, and other infectious symptoms, with stools presenting as watery, mucoid, or bloody mucoid. CDI can be classified by severity into mild to moderate CDI, severe CDI, severe or complicated CDI, and recurrent CDI [12].

Metronidazole and vancomycin have been the first-line drugs for treating CDI for several decades [13]. The American College of Gastroenterology recommends metronidazole for treating initial mild-to-moderate CDI in adults, while oral vancomycin is suggested for severe and complicated CDI patients (500 mg every 6 h). In cases where oral medication cannot reach the affected site, such as with intestinal obstruction, ileostomy, or colostomy, concurrent vancomycin enema administration is recommended until symptoms resolve [14, 15]. However, due to the difficulty in eradicating CDI and its high recurrence rate, fecal microbiota transplantation (FMT) has gained considerable attention and research in recent years for CDI treatment, and the effectiveness and safety of FMT treatment have gradually been recognized in clinical practice [16, 17]. FMT involves transplanting feces from a healthy donor into the patient’s gastrointestinal tract to treat specific diseases related to changes in the gut microbiota [18]. Research has shown that FMT has an effectiveness rate of over 80% in treating recurrent CDI, significantly higher than traditional treatments based on oral metronidazole [19]. Moreover, FMT has demonstrated excellent safety, with the European Society of Clinical Microbiology and Infectious Diseases and the American College of Gastroenterology recommending its use for severe or recurrent CDI [20].

Reviewing this case, the patient had multiple risk factors for CDI, including advanced age, colectomy with end ileostomy, food-induced intestinal obstruction, antibiotic use, and weakened immune function. However, due to the increased ileostomy output in the early stages of clostridium difficile infection (CDI), which is difficult to distinguish from the normal post-surgery condition, timely identification and appropriate treatment were not provided, leading to prolonged hospitalization. In the later stages of infection, after undergoing ileostomy closure surgery, the patient displayed typical CDI symptoms, such as high-volume diarrhea, fever, and elevated PCT levels. Stool culture confirmed clostridium difficile infection, followed by oral vancomycin and FMT treatment, along with supportive measures such as intravenous fluid replacement, oral Lactobacillus acidophilus, glutamine granules, guar gum, and probiotics, resulting in gradual improvement and discharge.

This case suggests that rectal resection and ileostomy, as risk factors for CDI, patients with a significantly increased and persistent output following ileostomy should be alert to the possibility of CDI and perform stool pathogen testing early to allow for timely, targeted treatment. For patients with poor oral medication response, FMT therapy can be considered.

## Data Availability

No datasets were generated or analysed during the current study.

## Declarations

### Credit for reproduced images

All reproduced images appearing in the manuscripts have been consented to by the patient, the leading doctor, and the hospital.

### The statements of approval

The cases reported in this article have been approved by the patient, the leading doctor, and the hospital. The approval PDF file has been uploaded to the related file.

## Abbreviations

CRC: Colorectal cancer
CD: Clostridium difficile
CDI: clostridium difficile infection
FMT: fecal microbiota transplantation
